# Genome Sequences of Alkalihalobacillus clausii, Bacillus safensis, Escherichia coli, and Pasteurella multocida Isolates from the Rumen, Nasopharynx, Vagina, and Uterus of Healthy Beef Cattle

**DOI:** 10.1128/mra.01274-22

**Published:** 2023-02-13

**Authors:** Gabriela Magossi, Devin B. Holman, Katherine E. Gzyl, Kaycie N. Schmidt, Scott A. Hoselton, Samat Amat

**Affiliations:** a Department of Microbiological Sciences, North Dakota State University, Fargo, North Dakota, USA; b Lacombe Research and Development Centre, Agriculture and Agri-Food Canada, Lacombe, Alberta, Canada; University of Maryland School of Medicine

## Abstract

Here, we present the first draft genome sequences of 10 bacterial strains that were isolated from the rumen, nasopharynx, vagina, or uterus of healthy beef cattle. These genomes are from one Alkalihalobacillus clausii isolate, three Bacillus safensis isolates, five Escherichia coli isolates, and one Pasteurella multocida isolate.

## ANNOUNCEMENT

Ten bacterial strains were isolated from ruminal fluid and deep nasopharyngeal, vaginal, and uterine swab samples collected from Angus-based steers, pregnant and nonpregnant heifers, and cows born and raised at the Central Grasslands Research Extension Center (Streeter, ND, USA). Samples were collected from the nasopharynx, rumen, and vagina as described previously (1). The uterine sampling was performed using a double-guarded culture swab (71-cm-long swab; Reproduction Provisions LLC, Walworth, WI, USA), which was guided via rectal palpation through the vagina to the cervix; the inner plastic portion was then threaded through the cervix and into the uterine body. Once in the uterine body, the swab tip was extended through the inner plastic sheath, making contact with the inner wall of the uterine body, and was gently swirled around several times. After sample collection, the swab was retracted into the inner and outer plastic sheaths and removed from the cow.

Isolates were cultured by plating the fresh ruminal fluid samples and the nasopharyngeal, vaginal, and uterine swab samples onto tryptic soy agar supplemented with 5% defibrinated sheep blood (BD, Franklin Lakes, NJ, USA) and incubating the plates overnight at 37°C with 5% CO_2_. For genomic DNA extraction, each cryopreserved isolate was regrown in brain heart infusion broth (BD) overnight at 37°C with 5% CO_2_, and 1 mL of this culture was centrifuged at 8,000 × *g* for 10 min at 4°C. DNA was then extracted from the cell pellet using the DNeasy blood and tissue kit (Qiagen, Inc., Hilden, Germany), with modifications as detailed in our previous publication (1), and the concentration was determined using a Qubit 4 fluorometer (Thermo Fisher Scientific, Waltham, MA, USA) with a double-stranded DNA (dsDNA) high-sensitivity (HS) assay kit (Thermo Fisher Scientific). An FX DNA library preparation kit (Qiagen) was used to prepare genomic libraries, which were then sequenced on an Illumina MiSeq instrument with the MiSeq v.2 reagent kit (500 cycles; Illumina, Inc., San Diego, CA, USA), following the manufacturer’s instructions.

Sequences were quality filtered using fastp v.0.23.2 (2) with automatic adapter trimming, and reads were discarded if they were <100 bp in length or had a mean Phred quality score of <15 with a sliding window of 4 bp. The paired-end reads were assembled with SPAdes v.3.15.5 (3) using the isolate option. Contigs of <500 bp in length were removed with BBMap v.38.96 (https://sourceforge.net/projects/bbmap) with the minlength parameter set to 500 bp. Completeness and contamination were calculated with CheckM v.1.2.0 (4) using the lineage-specific workflow, and the assembly quality was assessed with QUAST v.5.2.0 (5). Taxonomy for each bacterial isolate was assigned with GTDB-Tk v.2.1.1 (6) using the classify workflow and the Genome Taxonomy Database (GTDB) release 207 (7). Assemblies were annotated by NCBI using the Prokaryotic Genome Annotation Pipeline (PGAP) v.2022-10-03.build6384 (8). Default parameters were used for all software unless otherwise specified. The assembly statistics and number of coding sequences for each assembled genome are shown in Table 1.

**TABLE 1:** Sequencing summary and basic properties of Alkalihalobacillus clausii (*n* =1), Bacillus safensis (*n* = 3), Escherichia coli (*n* = 5), and Pasteurella multocida (*n* =1) strains isolated from the bovine rumen, nasopharynx, vagina, or uterus

BioSample accession no.	Strain identification no.	Species	Isolate origin	GenBank assembly accession no.	SRA accession no.	No. of contigs	No. of reads	Genome size (bp)	*N*_50_ (bp)	Avg coverage (×)	Total no. of coding sequences	G+C content (%)
SAMN31874034	2RFP1A2	Escherichia coli	Ruminal fluid	JAPNOB000000000.1	SRR22456541	133	1,615,854	5,033,318	103,059	161	4,872	50.5
SAMN31874035	2RFP1C2	Escherichia coli	Ruminal fluid	JAPNOC000000000.1	SRR22456540	106	722,606	5,245,691	168,359	69	5,077	50.5
SAMN31874036	2RFP1E	Alkalihalobacillus clausii	Ruminal fluid	JAPNOD000000000.1	SRR22456539	94	798,796	4,443,298	110,691	90	4,405	44.7
SAMN31874037	2RFP1H	Escherichia coli	Ruminal fluid	JAPNOE000000000.1	SRR22456538	134	784,614	4,855,413	105,516	81	4,658	50.6
SAMN31874038	2RFP8A2	Bacillus safensis	Ruminal fluid	JAPNOF000000000.1	SRR22456537	13	1,252,198	3,672,327	942,530	170	3,722	41.6
SAMN31874039	3RFP5C	Bacillus safensis	Ruminal fluid	JAPNOG000000000.1	SRR22456536	20	1,789,660	3,655,693	953,358	245	3,680	41.6
SAMN31874040	3RFP5E	Bacillus safensis	Ruminal fluid	JAPNOH000000000.1	SRR22456535	18	1,517,272	3,782,685	1,021,124	201	3,873	41.6
SAMN31874041	PM785D1B	Pasteurella multocida	Nasopharynx	JAPNOI000000000.1	SRR22456534	53	1,653,682	2,287,082	118,178	362	2,131	40.2
SAMN31874042	TP267CBC	Escherichia coli	Vagina	JAPNOJ000000000.1	SRR22456533	150	772,296	5,118,492	126,633	75	4,993	50.2
SAMN31874043	TP1813CBA	Escherichia coli	Uterus	JAPNOK000000000.1	SRR22456532	131	944,282	5,121,541	177,924	92	4,989	50.5

### Data availability.

All raw genome sequences and draft genome assemblies have been deposited in the Sequence Read Archive (SRA) and GenBank, respectively, under the accession numbers listed in Table 1.
